# Integrating force and position: testing model predictions

**DOI:** 10.1007/s00221-016-4734-1

**Published:** 2016-07-23

**Authors:** Femke E. van Beek, Wouter M. Bergmann Tiest, Astrid M. L. Kappers, Gabriel Baud-Bovy

**Affiliations:** 1 Department of Behavioural and Human Movement Sciences, MOVE Research Institute Amsterdam, Vrije Universiteit Amsterdam, Amsterdam, The Netherlands; 2 Department of Robotics, Brain and Cognitive Sciences (RBCS), Istituto Italiano di Tecnologia, Genoa, Italy; 3 Faculty of Psychology, Universitá Vita-Salute San Raffaele, Milan, Italy; 4 Experimental Psychology Unit, Ospedale San Raffaele, Milan, Italy

**Keywords:** Haptic perception, Psychophysics, Force field, Bisection model, Sensory integration

## Abstract

In this study, we investigated the integration of force and position information in a task in which participants were asked to estimate the center of a weak force field. Two hypotheses, describing how participants solved this task, were tested: (1) by only using the position(s) where the force reaches the detection threshold, and (2) by extrapolating the force field based on perceived stiffness. Both hypotheses were also described formally, assuming a psychophysical function obeying a power law with an exponent smaller than one. The hypotheses were tested in two psychophysical experiments, in which 12 participants took part. In Experiment 1, an asymmetric force field was used and the presence of visual feedback about hand position was varied. In Experiment 2, a unilateral force field was used. For both experiments, hypothesis 1 predicts biases between (Experiment 1) or at (Experiment 2) the position(s) of the force detection threshold, while hypothesis 2 predicts smaller biases. The measured data show significant biases in both experiments that coincide with the biases predicted by using force detection thresholds from the literature. The average measured responses and their variabilities also fitted very well with the mathematical model of hypothesis 1. These results underline the validity of hypothesis 1. So, participants did not use a percept of the stiffness of the force field, but based their estimation of the center of the force field on the position(s) where the force reached the detection threshold. This shows that force and position information were not integrated in this task.

## Introduction

Even though we perceive the world through many different sensory modalities, which provide us with different types of information about the world, we still have only one percept of the world. Recently, there has been a lot of attention in the perceptual literature on how we deal with all these different sources of information. A very influential idea is the maximum likelihood estimation model, which describes the integration of different sensory estimates as a statistically optimal integration, influenced by prior knowledge of the world (Ernst and Banks 2002; Van Beers et al. 1999). This idea can be applied to the integration of information across modalities (Ernst and Bülthoff 2004) or across different types of information within the same modality (Jacobs 1999; Landy et al. 1995). In our study, we investigated the integration of information within the haptic modality.

A striking example of the combination of haptic cues is a study by Robles-De-La-Torre and Hayward (2001), in which participants were asked to discriminate between holes and bumps. Even if the geometrical properties signaled a bump, a change in the force cues could cause the bumps to be perceived as holes. In a comparable experimental paradigm, Drewing and Ernst (2006) showed that weights of the force and position cues in curvature perception depend on the type of shape that is being explored: when the shape is strongly curved, position cues are weighted more strongly, while force cues are weighted more strongly when the shapes are flatter. In our study, we were also interested in the integration of force and position cues within the haptic modality. Specifically, we investigated the integration of force and position information in the haptic perception of the center of a weak elastic force field.

This question is particularly interesting when the force field of interest is very weak, as the forces already become imperceptible at some distance from the true center of the force field. The threshold for correctly discriminating between a right- and leftward force is  0.1 N in a static situation and  0.05 N when movement is allowed (Baud-Bovy and Gatti 2010), so as soon as forces exceed these values at some distance from the center only, humans somehow have to infer the true center of the force field. By studying these weak force fields, we can learn something about how humans combine position and force information over time, as they have to acquire the information by exploring the force field. In this study, we tested two hypotheses, which were: (1) participants do not use stiffness information, but only use the position at which the force becomes imperceptible, which we call the bisection model, and (2) participants estimate the stiffness of the force field through exploration. By extrapolating the forces based on the estimated stiffness, they estimate the position of the center of the force field, which we call the stiffness model. The former model has been described previously (Baud-Bovy 2014; Bocca and Baud-Bovy 2009).

If hypothesis 2 is correct, participants use stiffness information, which they gathered during the exploration of the force field. It is not known yet which parameters we use to perceive stiffness. Jones and Hunter (1990) found the discrimination threshold of stiffness to be higher than those of force and position, but they did show that participants used both force and position cues in their stiffness discrimination task. Tan et al. (1995) underlined the relevance of work cues, while Srinivasan and LaMotte (1995) suggested that the rate of change of average pressure might play an important role. There is also a considerable amount of work on the integration of visual and haptic information in stiffness perception (e.g., Korman et al. 2012; Kuschel et al. 2010). Irrespective of what parameters are being used to estimate stiffness, all theories share the fact that information needs to be acquired in a serial fashion, since stiffness is meaningless in a static situation. So, hypothesis 2 implies that participants acquire information over time by exploring the force field. By doing this, they build a percept of the stiffness of the force field and use the product of all this information to extrapolate the center of the force field. Importantly, this hypothesis also implies that position and force information need to be integrated, in order to obtain an estimate of the stiffness. So, if this hypothesis is true, integration between force and position information takes place in this task.

If hypothesis 1 is correct, the nature of the task is less sequential than in the case of hypothesis 2. Hypothesis 1 assumes that by exploring the force field, participants find the position(s) where the force just reaches the detection threshold. In the case of a bilateral force field (present in Experiment 1), they assume the center of the force field to be in between the two positions where the force reaches the threshold, while for a unilateral force field (present in Experiment 2), they assume the center of the force field to be at the position where the force reaches the threshold. If this is true, knowing the force threshold and the stiffness of the force field is enough to predict the position which participants will perceive to be the center of the force field. So, if hypothesis 1 is true, participants do not integrate force and position information to obtain a percept of the stiffness in this task, even though they do explore the force field and thus could have access to this information.

We designed two experiments to test the validity of the hypotheses. In Experiment 1, an asymmetric force field was used. In Experiment 2, a unilateral force field was used. For both experiments, hypothesis 1 (the bisection model) predicts biases between (Experiment 1) and at (Experiment 2) the force detection threshold, while hypothesis 2 (the stiffness model) predicts smaller biases. Moreover, in Experiment 1, the visual feedback was manipulated, which was predicted to influence the noise in the responses. Both experiments can differentiate between the two hypotheses and can thus contribute to testing if the brain integrates force and position information in this task.

**Fig. 1:**
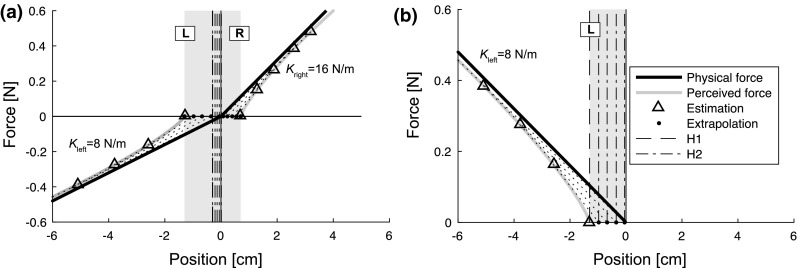
Illustration of the predictions from the 2 hypotheses for different types of force fields. The *vertical gray area* indicates the area of the workspace in which the force is below threshold level and thus imperceptible to the participant. The width of this area depends on the spring stiffness (*K*) of the force field, which is indicated with the *black line*. **a** For an asymmetric bilateral force field, the bisection model predicts a shift of the end position toward the weaker force field (*dashed line*, *H1*), in the middle of the right (*R*) and the *left* (*L*) positions of the force threshold. The stiffness model predicts a bias in the same direction, but a weaker one. For this figure, it is assumed that the stiffness estimates at both sides of the force field are made at the same force magnitude (the *triangles* show the positions and forces at which the estimates are made), from which the edges of the force fields are extrapolated, as shown with the *black dots*. The middle between the black dots is the prediction of the estimated center of the force field from the stiffness model (*dashed-dotted line*, *H2*). This results in a decrease in bias with an increase in position at which the stiffness estimate is made, up to biases disappearing for positions at the edge of the workspace. For both hypotheses, the direction of the predicted bias does not depend on the exact values of the spring stiffnesses ($K_{{{\mathrm {left}}}}$ and $K_{{{\mathrm {right}}}}$), but only on which spring is weaker. **b** In a unilateral force field, only one spring is present, so the right half of the figure is empty. The bisection model predicts an end position at the force threshold (*dashed line*, *H1*). It also predicts larger biases for weaker springs, since the vertical gray area is larger in that case. The stiffness estimation hypothesis predicts weaker biases (*dashed–dotted line*, *H2*), up to biases disappearing completely when stiffness is estimated using the largest forces in the force field

## Models

In this section, the mathematical descriptions of the bisection model and the stiffness model are explained. Both models are used to describe the behavior of participants who are asked to find the center of a force field in a one-dimensional situation (i.e., along a line). Two situations are described in particular: (1) a bilateral force field, in which one spring is active in the region left of the central position of the force field and the other is active in the region to the right of the center of the force field. These springs can be different, creating an asymmetric force field, and (2) a unilateral force field, in which only one spring is present. In this case, the term ‘center of the force field’ is used to indicate the point where the spring is attached, so the position along the line where the force just becomes 0. Both models can predict the bias in finding the center of a force field. These values can also be determined experimentally, which allows for a comparison between the validity of the two models based on the experimental data. In Fig. 1, a visual explanation of the predictions of these models for finding the center of a force field is given. For both models, a stationary reference frame is used, of which the origin is positioned at the center of the force field. To be able to predict the biases, the relation between the physical and estimated (or perceived) force magnitude needs to be known. We assume that the physical (*F*) and perceived ($\tilde{F}$) force are related through a power function (Stevens and Marks 1999; Stevens 1957):

1 $${ \tilde{F} = \alpha (F - F_{{{\mathrm {th}}}}) ^{\beta }.}$$

In this equation, $F_{{{\mathrm {th}}}}$ is the force threshold above which the relation exists, while $\alpha$ and $\beta$ determine the shape of the relation. In our previous experiments, we measured force perception for a range of forces using a haptic device. In those experiments, we found a mean $\beta$ of 0.8 (Van Beek et al. 2015), so we will assume this $\beta$ for the current experiment too. In a linear force field, the force is dependent on the spring constant (*K*) and the position (*x*), so we can describe Eq. 1 in the following way in this situation:

2 $${ \tilde{F} = \alpha (K (x-x_{0}))^{\beta }}$$

with $x_{0}$ being the position where the perceived force is 0. Note that for Experiment 2, a minus sign needs to be added before *K*, since the force field is an attractive one in this experiment. Starting from Eq. 2, participants could use 2 approaches to find the center of the force fields: by only using the position(s) where the perceived force just reaches 0 (i.e., the bisection model, which is hypothesis 1), or by probing the relation between position and force and extrapolating the position where the force becomes 0 (i.e., the stiffness model, which is hypothesis 2). The models describing the two hypotheses are explained in more detail below.

### Bisection model

The bisection model was first proposed in Bocca and Baud-Bovy (2009), while an extended version is described in detail in Baud-Bovy (2014). The model is based on the idea that the participant does not use stiffness information, but only uses the position(s) where the force reaches the threshold. In the model, parameters describing the force threshold and the perception of hand position are used. Furthermore, a weight factor between the left and right positions where the force reaches the threshold is added. These parameters are combined to describe the bias and the variability in the measured data. For the bias, Baud-Bovy (2014) used the following:

3 $$\mu _{x} = (1 - w) \frac{-F_{{{\mathrm {th}}}}}{K_{{{\mathrm {left}}}}} + w \frac{F_{{{\mathrm {th}}}}}{K_{{{\mathrm {right}}}}}.$$

So the estimation of the measured bias, $\mu _{x}$, is based on the mean position between the two positions left and right of the center where the force reaches the threshold. These positions depend on the value of the force threshold, $F_{{{\mathrm {th}}}}$, and the stiffness of the force field that is used, which is represented by the stiffness of the left spring, $K_{{{\mathrm {left}}}}$, and the right spring $K_{{{\mathrm {right}}}}$. Note that *K* in this equation is a value that is only used to be able to make predictions, and it is not a parameter that participants need to estimate, since they only need to estimate their hand position. In contrast, the value of $\tilde{K}$ in the stiffness model is a parameter of which participants do need to make an estimate.

The factor *w* is a weight factor between the two positions, which was introduced in the model in Baud-Bovy (2014), mainly because the device was not positioned at the body midline in their experiment, which appeared to cause a shift in their data. In our experiment, the device was positioned at the body midline, so there was no reason to expect a difference in weight between the left and the right positions. Therefore, *w* was fixed at $\frac{1}{2}$ in the current experiment, resulting in:

4 $$\mu _{x} = \frac{1}{2} \left( \frac{-F_{{{\mathrm {th}}}}}{K_{{{\mathrm {left}}}}} + \frac{F_{{{\mathrm {th}}}}}{K_{{{\mathrm {right}}}}} \right) .$$

To describe the within-participant variability between the trials, the following equation was used:

5 $$\sigma _{x}^2 = (1 - w)^2 \left( \frac{\sigma _{F_{{{\mathrm {th}}}}}^2}{K_{{{\mathrm {left}}}}^2} + \sigma _{P}^2 \right) + w^2 \left( \frac{\sigma _{F_{{{\mathrm {th}}}}}^2}{K_{{{\mathrm {right}}}}^2} + \sigma _{P}^2 \right) .$$

The within-participant variability is $\sigma _{x}$, while $\sigma _{F_{{{\mathrm {th}}}}}$ and $\sigma _{P}$ describe the variability in the force threshold and the perception of hand position, respectively. The assumption in the model is that the variability in both signals is combined optimally, which is done mathematically by adding the variances, resulting in a final prediction of the measured variability. Again, *w* was fixed at $\frac{1}{2}$ in the current experiment, resulting in:

6 $$\sigma _{x}^2 = \frac{1}{4} \left( \frac{\sigma _{F_{{{\mathrm {th}}}}}^2}{K_{{{\mathrm {left}}}}^2} + \frac{\sigma _{F_{{{\mathrm {th}}}}}^2}{K_{{{\mathrm {right}}}}^2} + 2 \sigma _{P}^2 \right) .$$

In one of our experiments, we used two types of visual feedback about hand position to assess its effect on the positional noise parameter. To be able to fit the data of both of the visual feedback conditions together to make sure that as few parameters as possible were used, Eq. 6 was modified to:

7 $$\sigma _{x}^2 = \frac{1}{4} \left( \frac{\sigma _{F_{{{\mathrm {th}}}}}^2}{K_{{{\mathrm {left}}}}^2} + \frac{\sigma _{F_{{{\mathrm {th}}}}}^2}{K_{{{\mathrm {right}}}}^2} + 2 k \sigma _{P_{{{\mathrm {fp}}}}}^2 + (1-k) 2 \sigma _{P_{{{\mathrm {fa}}}}}^2 \right) .$$

We assumed the visual feedback to only affect the positional noise parameter, since that was the only parameter that the visual feedback provided any information about. Therefore, we included two position parameters, while fitting the same bias, force threshold and noise of the force threshold for all data. The positional noise for the condition in which visual feedback was present was $\sigma _{P_{{\mathrm {{fp}}}}}$, while the noise for the condition in which visual feedback was absent was $\sigma _{P_{{\mathrm {{fa}}}}}$. The value of *k* was used as a switch: For the condition in which visual feedback was present, *k* was 1, and for the condition without visual feedback, it was 0.

If the force field is unilateral, so only one spring is present, the bisection model can be simplified to:

8 $$\mu _{x} = \frac{F_{{{\mathrm {th}}}}}{K}$$

for the bias, and

9 $$\sigma _{x}^2 = \frac{\sigma _{F^2}}{K^2} + \sigma _{P}^2$$

for the variability. In the fitting procedure, a least-squares approximation was used, in which the sum of squares (SS) was minimized for the bias and variability together:

10 $${{\mathrm {SS}}} = \sum _{i=1}^{n} \left( x_{i} - \mu _{x_{i}} \right) ^2 + \left( s_{i} - \sigma _{x_{i}} \right) ^2.$$

In this equation, *i* refers to the number of the condition, while *n* is the total number of conditions. The measured bias in the *i*-th condition is $x_{i}$, while the measured variability is $s_{i}$. The bias and variability derived from the model for that condition are $\mu _{x_{i}}$ and $\sigma _{x_{i}}$, respectively. This fitting procedure can be used for both experiments and both models.

### Stiffness model

In the stiffness model, it is assumed that participants estimate the stiffness of the force field by exploring it. When they have formed this estimate, they can use it to find the distance from their current position to the position where the force becomes 0 by extrapolating the current force, as long as it is above the force threshold, through:

11 $$\tilde{x_{d}} = -\frac{\tilde{F}}{\tilde{K}}.$$

In this equation, $\tilde{x_{d}}$ is a signed quantity that indicates the movement needed to reach the edge of the force field, while $\tilde{F}$ is the force sensed at the current hand position, and $\tilde{K}$ is the estimated stiffness. Note that for Experiment 2, the force field is reversed, so to describe that situation, the minus sign before $\tilde{F}$ needs to be removed. When combining this equation with Eq. 2, we obtain:

12 $${ \tilde{x_{d}} = -\frac{\alpha ( K (x-x_{0})^{\beta })}{\tilde{K}}.}$$

We assume that the estimated stiffness corresponds to the slope of the psychophysical function:

13 $$\tilde{K} = \frac{\hbox {d}\tilde{F}}{\hbox {d}\tilde{x}} = \frac{\hbox {d}\tilde{F}}{\hbox {d}x}\frac{\hbox {d}x}{\hbox {d}\tilde{x}}.$$

Since we assume the position signal to be unbiased, the last term equals 1 and thus disappears. By now combining Eqs. 2 and 13, we obtain:

14 $${ \tilde{K} = \frac{\hbox {d}}{\hbox {d}x} \alpha (K (x-x_{0}))^{\beta } = K \beta \alpha (K (x-x_{0}))^{\beta -1}. }$$

By now combining Eqs. 12 and 14, we obtain a new description of $\tilde{x_{d}}$:

15 $${ \tilde{x_{d}} = - \frac{\alpha (K (x-x_{0}))^{\beta }}{K \beta \alpha (K (x-x_{0}))^{\beta - 1}} = - \frac{(x-x_{0})}{\beta }.}$$

If the position (*x*) of the handle changes, the perceived force ($\tilde{F}$) changes and thus $\tilde{x_{d}}$ changes. This allows for multiple estimates ($\tilde{x_{c}}$) of the edge of the same force field, through:

16 $${ \tilde{x_{c}} = x + \tilde{x_{d}} = x - \frac{(x-x_{0})}{\beta }.}$$

So, in this model, the estimated center of the force field depends on the current position, the position at which the perceived force becomes 0, and $\beta$. The final bias in a bilateral force field must be based on an integration of the extrapolations at the 2 sides, through:

17 $${ \tilde{x_{c}} = \frac{1}{2} (\tilde{x_{c_{{\mathrm {left}}}}} + \tilde{x_{c_{{\mathrm {right}}}}}).}$$

By comparing the perceived and the actual position of the center of the force field, the bias can be obtained:

18 $$\mu _{x} = \tilde{x_{c}} - x_{c}.$$

As the actual position of the center of the force field is placed at position 0, Eqs. 16, 17, and 18 can be combined to describe the bias:

19 $${ \mu _{x} = \frac{1}{2} \left( (x_{{\mathrm {left}}} - \frac{x_{{\mathrm {left}}}-x_{0_{{\mathrm {left}}}}}{\beta }) + (x_{{\mathrm {right}}} - \frac{x_{{\mathrm {right}}}-x_{0_{{\mathrm {right}}}}}{\beta }) \right) }$$

which can be simplified to:

20 $${ \mu _{x} = \frac{1}{2} \left( x_{{\mathrm {left}}} + x_{{\mathrm {right}}} - \frac{x_{{\mathrm {left}}} + x_{{\mathrm {right}}} - x_{0_{{\mathrm {left}}}} - x_{0_{{\mathrm {right}}}}}{\beta } \right) . }$$

To qualitatively describe this situation, Fig. 1 is made. From this figure, it is clear that the estimation of the position of the center of the force field in the stiffness model is closer to the true center of the force field than in the bisection model for both experiments. However, the precise position of the estimation depends on the hand position at which the estimation is made in the stiffness model, which is unknown. So, we can only obtain a quite large range of predictions, and not one value. Therefore, our approach will be as follows: we will first predict the biases using the bisection model, while taking force detection thresholds from the literature [0.05–0.1 N, as reported in Baud-Bovy and Gatti (2010)]. If the measured biases are smaller than the predicted ones, this suggests the stiffness model describes the data more appropriately. In that case, we will make additional assumptions about hand position to be able to fit the stiffness model, which will also allow us to make predictions about the variability of the data. If we do obtain force thresholds that fit the predicted values, the stiffness model cannot explain the data, and we proceed with fitting the bisection model only. In the latter situation, the bisection model will be fitted to the data with the force detection threshold being a free parameter, so the fitted detection threshold is also expected to lie between 0.05 and 0.1 N.

## Material and methods

### Participants

In Experiment 1, 12 participants took part, 2 females and 10 males. They were $31\pm 6$ years old; 10 were right-handed, and 2 were left-handed. In Experiment 2, 12 other participants took part, 3 females and 9 males. They were $34\pm 14$ years old, and all were right-handed. None of the participants had a history of neurological disorders. All participants signed an informed consent form and were given written instructions prior to the experiment, while being naive to the purpose of the experiment. Both experiments were approved by the local ethics committee.

### Setup

**Fig. 2:**
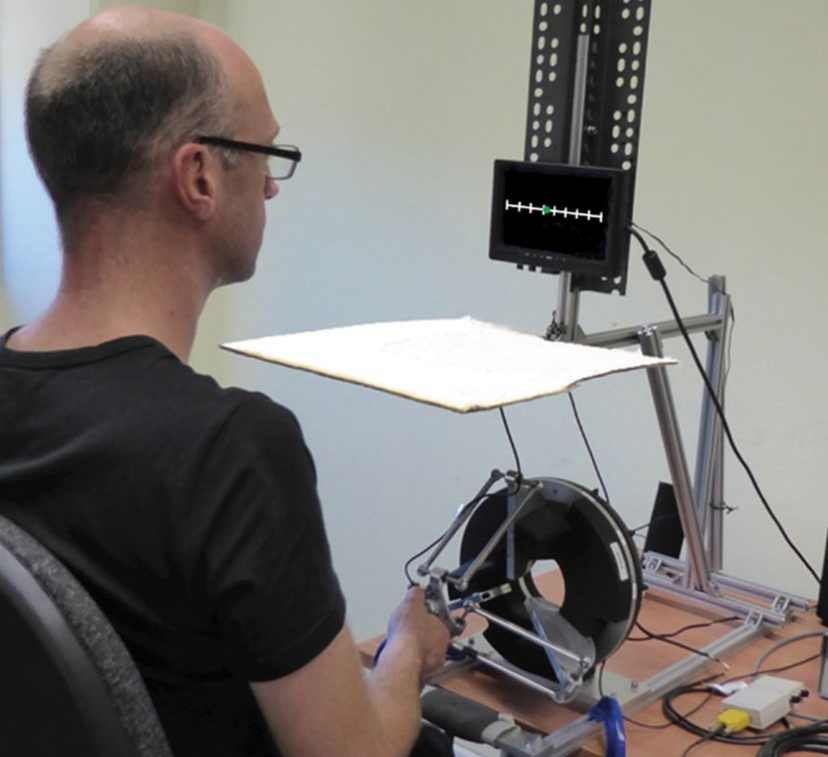
Participant holding the handle of the Omega.3. The device is restricted to movements along a line parallel to the frontal axis of the participant, so participants can only make left–right movements. The response box, which is normally held in the non-dominant hand, is lying on the table in the lower right corner of the picture. On the screen, the feedback presented in Experiment 1 is shown: The position of the dot on the line indicates the participant’s hand position, while the green color indicates that the grip force is within the required range

Both experiments were performed using the Omega.3, as shown in Fig. 2 (Force Dimension, Switzerland). This is an impedance-controlled haptic device, with an end effector with three translational degrees of freedom. In this experiment, the movement of the device was restricted to one dimension, which was in the horizontal plane, parallel with the frontal axis of the participant. Thus, participants could only make left–right movements along a line of 20 cm. The middle of the device was aligned with the body midline of the participant. The gravity of the device was compensated using the Force Dimension DHD antigravity compensation scheme, with a mass parameter of 0.06 kg. To provide higher transparency, a closed-loop force control law was implemented in the device by adding a force sensor at the end effector. This customization was performed previous to our experiment, and is described in detail in Gurari and Baud-Bovy (2014). The control loop was implemented as follows:

21 $$f_{{{\mathrm {cmd}}}} = f_{d} - k_{f} (f_{m} - f_{d}).$$

In this control loop, $f_{{{\mathrm {cmd}}}}$ is the force command sent to the motors, $f_{m}$ is the force measured by the force/torque sensor, $f_{d}$ is the desired force based on the desired elastic force field, and $k_{f}$ is the feedback gain, which was 10 in our experiment. The measured force ($f_{m}$) was passed through an exponential filter with a time constant of 0.004 s. The force command ($f_{{{\mathrm {cmd}}}}$) was updated with a frequency of 1 kHz. Another customized feature was the addition of two pressure sensors to the handle, to measure the participant’s grip force. For more information about the customization of the device, see Gurari and Baud-Bovy (2014).

The participants always used their dominant hand to hold the handle of the haptic device, while they pressed the button on a response box with their other hand. To ensure a comfortable arm position, the chair height was adjusted to position the lower arm in the horizontal plane. Above the haptic device, at eye height, a small screen (Lilliput UK, 7”) provided visual information about grip force in all experiments. In Experiment 1, the screen was also used to provide visual feedback about hand position in one of the conditions. Vision of the hand was removed by placing a horizontal occluder below the feedback screen.

### Protocol

In both experiments, the task for the participants was to find the position in the force field where the force was 0. Each trial started with a homing phase, in which the device guided the participant to the start position of that trial (see below for a description of the pseudo-random selection of start positions). After the homing phase, the force field was applied gradually. When the force field was at complete strength, a beep indicated that the participants could start exploring the force field. When the participants felt they had reached the desired position, they pressed the button of the response box to confirm the position. Then, the experimenter started a new trial. There was no time limit, and participants were told that there was no need to respond as quickly as possible. To make sure that the participants kept a firm but gentle grip on the handle throughout the experiment, with a comparable grip force across participants, visual feedback of the grip force was provided through a dot on a screen. The color of the dot corresponded to the grip force: When grip force was within the range 0.25–1.5 N, the dot was green, while it turned red when the grip force was too high, and blue when it was too low. Participants were asked to make sure that the dot stayed green throughout each trial.

In Experiment 1, the elastic force field consisted of 2 linear springs, oriented right and left of the central position. The force field was oriented outwards, so a position farther from the central position corresponded to a stronger force away from the central position. This was done to avoid a ‘letting go’ strategy, in which participants would become so compliant that the machine would take them to the central position automatically. Because all the forces were fairly weak and the grip force was constantly monitored, this was not very likely, but we still wanted to avoid this situation. The task for the participants was to find the central position, which was described in the instructions as ‘the position where the force became 0’ or ‘the position where the force changed direction’. Both descriptions were given to the participants. In half of the trials, visual feedback of the hand position was provided, using a moving dot on a horizontal white line (14 cm), which corresponded to the complete workspace (20 cm). On the horizontal line, 10 equidistant vertical lines were placed to provide some visual anchors. In the trials with visual feedback, the position of the dot on the line, which was also the grip force feedback dot, was an accurate representation of the actual hand position. In the other half of the trials, the dot was placed at a random position along the line and was stationary throughout the trial. It is important to note that in none of the trials, the visual feedback provided any information about the position of the force field. In most conditions, the force field was asymmetric, so the pairs of springs had different stiffnesses. The asymmetric spring stiffness pairs used in the experiment (left spring and right spring) were: 16 and 4, 16 and 8, 8 and 4, 4 and 8, 8 and 16, 4 and 16 N/m. In one condition, both spring stiffnesses were 8 N/m, which was the only symmetric condition.

For each combination of spring stiffnesses, the central position of the force field was varied by using a random position in one of the three regions, which were defined with respect to the middle of the workspace: from −3 to −1 cm, from −1 to 1 cm and from 1 to 3 cm. The central position of the force field was chosen at a random position in each of the three regions. The start position of the device was chosen at a random position in a region between 1 and 3 cm from the central position of the force field. Each random position was used for 2 start positions: one at the random distance to the left of the force field and one at the same distance to the right of the force field. So, this resulted in 3 regions $\times$ 2 start sides = 6 trials per condition. We used 7 combinations of spring constants (6 asymmetric pairs and one symmetric force field) and 2 feedback conditions (feedback present and absent), resulting in 84 trials per participant. These trials were divided into 3 blocks, resulting in a total measurement time of maximally 1 h. In between the blocks, the participants could rest their arm to avoid fatigue.

In Experiment 2, the force field was unilateral, so only one linear spring was present. In this experiment, the force field was oriented inwards, so a position farther from the central position corresponded to a stronger force toward the central position. We could no longer use the instruction to ‘find the position where the force changed direction’. Instead, we presented the task by telling participants that there was a virtual object of which they had to find the edge. Since visualizing an object is easier when it pushes you out instead of dragging you in, we chose to invert the direction of the force field. By setting a minimum grip force level, participants could not ‘let go’ to find the center of the force field in Experiment 2 either. The instructions for Experiment 2 were to ‘to find the edge of the object, which pushes you out as soon as you enter it’ or ‘to find the position where the force just turns 0’. The spring constants were again 4, 8 or 16 N/m. The only visual feedback present in all the trials was the grip force dot, placed at a random horizontal position on the screen, while the vertical position was the same as in Experiment 1.

**Fig. 3:**
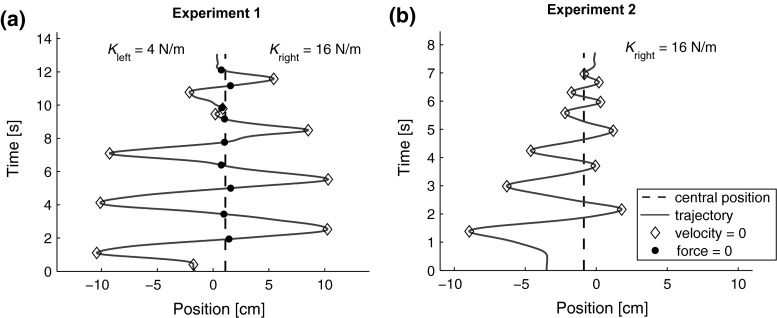
Typical examples of movement trajectories in both experiments. **a** Typical example of Experiment 1. The* diamonds* indicate a change in movement direction, at which the velocity is 0. The *black dots* indicate a change in force direction, at which the force is 0. The *dashed line* indicates the center of the force field, so the positions where the force is 0 are very close to this line. In this trial, the participant estimated the center of the force field to be left of the true center, since the trajectory ends left of the dashed line. This means that the bias is toward the weaker spring. **b** Typical example of Experiment 2. The dots are omitted in this graph, because the zero-force crossings are not well defined, since the force is 0 everywhere to left of the central position. In this trial, there was only a spring on the right side of the workspace (which is the positive direction). The end position shows a bias toward this direction, so the end position lies slightly inside the object

Again, we varied the position of the center of the force field by choosing random positions within the 3 defined regions. The start position of the device was chosen at a random position in a region between 1 and 3 cm from the central position of the force field and was always positioned outside of the object. We used 2 repetitions, resulting in 3 regions $\times$ 2 repetitions = 6 trials per condition. Since there were 6 conditions, the total number of trials was 36 per participant. The total experiment took about 20 min. Halfway into the experiment, the participants were asked whether they wanted to have a break. Some participants did take a break, while others completed the experiment in a single block. During the experiment, force and position data were recorded with a frequency of 250 Hz. Prior to both experiments, some practice trials were performed to familiarize the participants with the task.

For both experiments, the stiffness model and the bisection model give different predictions. These predictions are illustrated in Fig. 1. For Experiment 1, the bisection model (H1) predicts a bias towards the weaker force field, while the stiffness model (H2) predicts a range of biases, ranging from a smaller bias in the same direction to no bias at all, depending on the participant's hand position. For Experiment 2, the bisection model, which is actually a one-side model in this case, predicts that the participant perceives the edge of the force field to be positioned at the force threshold (H1). So, the bisection model predicts a bias towards the spring, resulting in a negative bias for a spring on the left side and a positive bias for a spring of the right side. The stiffness model again predicts a range of biases, ranging from a smaller bias than the one predicted by the bisection model to no bias at all (H2).

### Device performance

For both experiments, some general characteristics describing the performance of the device were calculated first. For a general impression of the type of movements participants made, see Fig. 3. An important measure of the performance of the device is the difference between the desired and the measured force. Since force feedback is never completely transparent, these measures are never completely the same. In Experiment 1, the median force error across participants was 0.0013 N. In Experiment 2, the median force error was 0.0018 N, so the errors were very well centered around 0. The median RMS of the force error was 0.021 N for both experiments, so the errors were also fairly small. The calculation of the RMS was only based on data acquired within the linear part of the force field, which was between −6 and 6 cm with respect to the center of the workspace. Another important measure is the position of the device at the zero-crossing of the force, as the position where the force changes direction is somewhat different in each movement. Moreover, because of friction and mechanical side effects, there is a slight difference in force feedback between moving to the right and moving to the left. As the task for the participants was to find the zero-position of the force, this measure is very relevant. In Experiment 1, the median standard deviation of the zero-crossings, averaged per trial and then across trials, was 0.32 cm. For Experiment 2, the zero-crossings were less well defined, since the desired force was zero everywhere outside of the object. So, for Experiment 2, the zero-crossings were not calculated. The small standard deviation of the zero-crossings shows that the desired and calculated central position of the force field matched closely. These measures together show that the performance of the device was good enough to be able to differentiate between the two different hypotheses, since biases were predicted to be in the range of about 0.5–3 cm.

### Data analysis

To first investigate the data qualitatively, some general characteristics to describe the movement patterns of the participants were calculated: movement time, total movement distance, number of times the movement direction was changed and movement distance between these changes. All measures were first calculated for each trial and then averaged across trials and participants. As there were large differences between participants in their movement strategy, the variability in these measures was very large and did not look like a Gaussian distribution. Therefore, the median of the data (and not the mean) will be presented to describe the results of the general characteristics across participants.

**Fig. 4:**
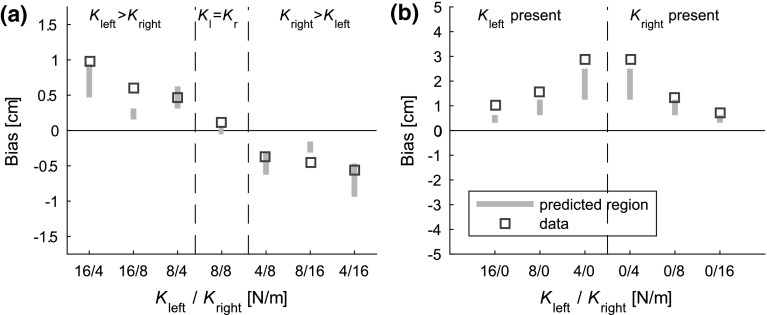
Measured data and biases predicted using the bisection model, based on force detection thresholds from the literature. The *squares* represent the mean of the measured biases (averaged across visual feedback conditions), while the *gray lines* represent the predictions from the bisection model for the range of force detection thresholds from the literature (0.05–0.1 N). **a** Measured data and predicted biases for Experiment 1. **b** Measured data and predicted biases for Experiment 2. If the measured biases were lower than the predicted ones, this would indicate that the stiffness model is the most likely explanation of the data. For both experiments, this is clearly not the case, so the bisection model is better at describing the data

The results for the end positions were more consistent across participants, so the results based on these data could be represented using mean ± standard error across participants. From the data sets of each participant, the bias and standard deviation were calculated for each condition. To calculate the bias, we used the difference between the central position and the end position of the movement, in which the latter was defined as the average position of the last 100 ms of the trial. The standard deviation was calculated across trials per condition and participant, so this represented the variability in the answers of each participant for each condition. For Experiment 1, for each trial, the positions at which the measured force changed direction were calculated, which we called zero-crossings. The mean position of these zero-crossing was used as the central position of the force field for that trial in further analyses, in order to use the best possible estimation of the force field that participants experienced, although the difference between the pre-defined and measured central position was very small. Since the zero-crossings were less well defined for Experiment 2, they were not used to determine the central position in this experiment, but the pre-defined central positions were used for further analyses.

An outlier analysis was performed on the basis of 2 criteria. Firstly, when participants used too much grip force or moved very fast, the setup sometimes produced high-frequency noise. When this happened for more than 0.5 s consecutively, the trial was rejected, which was the case for 6 trials (0.60 %) in Experiment 1 and 1 trial (0.23 %) in Experiment 2. The second criterion was based on the consistency of the data. For each condition, trials were rejected that were more than 5 SD away from the mean of the condition, when the mean was calculated without that particular trial. On the basis of this criterion, 10 trials (0.99 %) were rejected in Experiment 1 and 5 trials (1.2 %) were rejected in Experiment 2.

To assess the correspondence between predictions from the bisection model and measured biases, the biases were first predicted based on detection thresholds from the literature (0.05–0.1 N). The measured biases were averaged across visual feedback conditions for easy comparison with the predicted data. If the bisection model holds, the data should fall within the predicted range. If the measured biases were lower than the predicted ones, this would be in favor of the stiffness model. All further analyses and the fitting procedures were performed on data separated according to visual feedback condition.

To assess the effect of the different spring stiffnesses and of the visual feedback, repeated measures ANOVAs were performed, for the biases and the SD separately. When the sphericity criterion was not met, Greenhouse-Geisser correction was used. The biases and SD were averaged across participants before fitting a model. The goodness of fit of the model was assessed using an $R^2$ value.

**Fig. 5:**
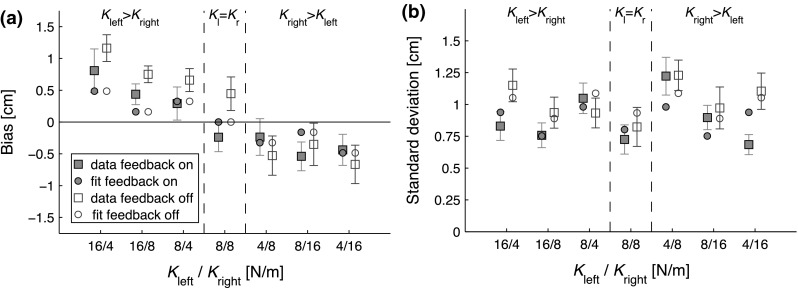
Data and fits of Experiment 1. The *squares* with *error bars* represent the mean ± one standard error, while the *dots* represent the fit of the bisection model to the data. On the horizontal axis, the stiffness pairs of the asymmetric force field are indicated. The *filled symbols* indicate the condition in which visual feedback is present, while the *open symbols* show the condition without visual feedback. **a** Biases of Experiment 1. All biases are oriented toward the weaker force field. Note the effect of combination of spring stiffnesses on the size of the bias. There was no significant effect of visual feedback type on the bias. **b** SD of Experiment 1. Note that the standard deviation is significantly higher for the condition without visual feedback, compared to the condition with visual feedback

## Results

### Measured and expected biases

To assess the validity of the models, the biases were first predicted based on detection thresholds from the literature (0.05–0.1 N, as reported in Baud-Bovy and Gatti (2010)). These predicted values are shown as gray bars in Fig. 4, as well as the measured data. For Experiment 1, the measured values mostly fall in the predicted range. The measured values in Experiment 2 are even a bit higher than expected. These observations are not in accordance with the stiffness model, so we did not use this model in the rest of the analysis. In the next subsections, the measured data and the fits made using the bisection model are discussed in more detail.

### Experiment 1

The performance of the participants was first characterized by investigating the movement trajectories qualitatively. Generally speaking, participants first made large sinusoidal movements, after which they gradually decreased their movement amplitude to ‘zoom in’ to the position where the center of the force field was located. A typical example of such a trajectory is given in Fig. 3a. Across all trials and participants, the median movement time was 10.9 s, while the total moved distance was 50.6 cm. During each trial, participants changed movement direction 11.1 times, while the median distance between two changes in direction was 5.9 cm.

The analysis of the endpoints showed biases toward the weak side of the force field, as shown in Fig. 5a. So, when the spring on the right (left) side was weaker, the biases were positive (negative) and thus rightwards (leftwards). Moreover, when the difference between the two springs was larger, the bias was also larger. The repeated measures ANOVA on the biases showed a significant main effect of force field stiffness ($F_{1.9,66}=12, \, \, p\le 0.001$), but not of visual feedback type ($F_{1,11}=1.5, \, \, p=0.25$). For the standard deviations, which are shown in Fig. 5b, the repeated measures ANOVA also showed a significant main effect of force field stiffness and a significant main effect of visual feedback type ($F_{6,66}=3.5, \, \, p=0.0043$ and $F_{1,11}=5.0, \, \, p=0.047$, respectively). So, even though there was no difference in bias between the two types of visual feedback, the standard deviation across trials within participants was smaller when the visual feedback dot was moving.

### Experiment 2

In this experiment, participants generally responded faster and made more asymmetric movements than in Experiment 1, as shown in the typical example in Fig. 3b. Their median movement time was 8.5 s, while the total moved distance was 39.2 cm. During each trial, participants changed movement direction 8.2 times, while the median distance between two changes in direction was 4.5 cm.

**Fig. 6:**
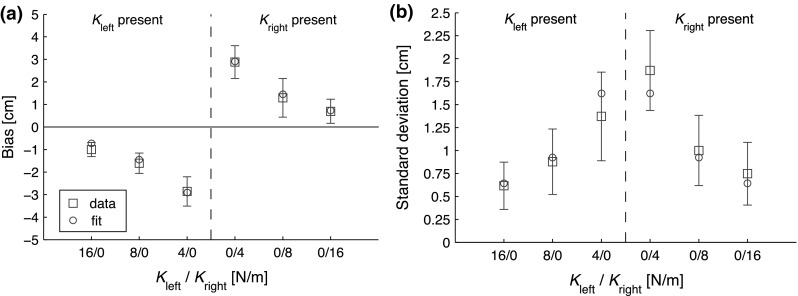
Data and fits of Experiment 2. The *squares* with *error bars* represent the mean ± one standard error, while the *dots* represent the fit of the bisection model to the data. On the horizontal axis, the stiffness of the unilateral force field is indicated (a 0 is noted for the side on which there was no spring). **a** Biases of Experiment 2. All biases are oriented toward the spring. Note that the bias increases for weaker springs. **b** standard deviations of Experiment 2. The standard deviations are also larger for weaker springs

The analysis of the endpoints showed significant biases, as can be seen in Fig. 6a. All mean biases were positive (negative), which means rightwards (leftwards), for a force field on the right (left). So, the participants perceived the center of the force field to be slightly inside the object. The repeated measures ANOVAs showed a significant main effect of type of force field on both the biases and standard deviations ($F_{5,55}=23.7, \, \, p<0.001$ and $F_{5,55}=9.93, \, \, p<0.001$, respectively). This is also reflected in Fig. 6: Both the biases and the standard deviations are larger for smaller force field stiffnesses.

**Table 1:** Fitted values using the bisection model, for Experiments 1 and 2

Experiment	$F_{{{\mathrm {th}}}}$ (N)	$\sigma _{F}$ (N)	$\sigma _{P_{{{\mathrm {fa}}}}}$ (cm)	$\sigma _{P_{{{\mathrm {fp}}}}}$ (cm)
1	0.085	0.052	1.2	0.93
2	0.12	0.062	0.51	(–)

### Model fits

The data of both experiments showed strong biases that were highly dependent on the stiffness of the force fields. Figure 4 illustrates that the measured data are not in accordance with the stiffness model, so only the fits of the bisection model are discussed in this subsection. For Experiment 1, the mean biases and SD were fitted together for both feedback conditions (See Eq. 10), while using the fitting parameters force threshold ($F_{{{\mathrm {th}}}}$), noise on the force threshold ($\sigma _{F}$)and noise on the position in the condition in which visual feedback was present ($\sigma _{P_{{{\mathrm {fp}}}}}$) and noise on the position in the condition in which visual feedback was absent ($\sigma _{P_{{{\mathrm {fa}}}}}$). This yielded a value of 0.085 N for the force threshold and 0.052 N for the noise on the force threshold. This force threshold falls exactly in the range of expected force thresholds (between 0.05 and 0.1 N), so this is not in accordance with the stiffness model.

The noise on the position data was lower for the condition in which visual feedback was present than for the condition in which it was absent. For the former condition, the fitted positional noise was 0.93 cm, while it was 1.2 cm for the latter. The goodness of fit was good, with an $R^2$ of 0.87.

For Experiment 2, the mean bias and standard deviations were also fitted together (see Eq. 10), while using the fitting parameters force threshold ($F_{{{\mathrm {th}}}}$), noise on the force threshold ($\sigma _{F}$), noise on the position ($\sigma _{P}$). This yielded values of 0.12 N for the force threshold and 0.062 N for the noise of the force threshold. The fitted force threshold is close to the range described in the literature, but it does not exactly match. However, if the stiffness model were true, thresholds beneath 0.05 N are expected, so the threshold being higher than the expected value is not in accordance with the stiffness model. The fitted noise on the position data was 0.51 cm. The goodness of fit was excellent, with an $R^2$ of 0.99. An overview of all the fit values is given in Table 1.

## Discussion

In both experiments, we found significant stiffness-dependent biases that were between (Experiment 1) or at (Experiment 2) the force detection threshold expected from the literature, which is in accordance with the hypothesis that participants base their estimation of the center of a force field on the position(s) where the force reaches the threshold level. If the stiffness model were used, much smaller biases would have been found. In Experiment 1, the biases were all oriented toward the weaker side of the force field. The values of the combination of spring stiffnesses significantly influenced both the biases and the standard deviation of the biases. This influence was also present in the model fits, as shown in the model fits in Fig. 5. The manipulation of visual feedback only significantly influenced the standard deviation and not the biases themselves, although the effect on the standard deviation was small. The bisection model also captured this feature, as it also produced a lower fit value for the positional noise in the condition in which visual feedback was present.

In Experiment 2, the biases were all oriented toward the force field, so all biases were positioned inside the virtual object. The stiffness of the force field significantly influenced both the biases and the standard deviations: a smaller stiffness caused larger biases and standard deviations. All these observations are in line with the predictions from the bisection model, while they do not support the prediction of biases smaller than the detection threshold from the stiffness model.

Together, these experiments not only provide strong evidence against the use of stiffness information in this task, but they also provide evidence that the bisection model is able to correctly predict biases and standard deviations for this task. So, it seems that participants indeed only use the position(s) where the force reaches the threshold to solve the task. The fitted values are comparable to values found in previous experiments using this model (Baud-Bovy 2014; Bocca and Baud-Bovy 2009). Moreover, the fitted force thresholds are very close to thresholds measured in other behavioral experiments (Baud-Bovy and Gatti 2010), even though our fitted thresholds are only based on the observed biases and standard deviations. It is puzzling that the positional noise in Experiment 2 is lower than that of both conditions of Experiment 1. An explanation could be that the integration between a left and a right position, which needs to be done in Experiment 1, adds some noise to the position estimate, which is currently not incorporated in the model. The force threshold is also a bit higher in Experiment 2. This could be caused by participants using a cautious strategy: it is safer to move a bit more inside the object when you want to be certain that you have localized it, which would result in an increase in the fitted force threshold. In Experiment 1, this strategy does not work, because there is a force field on both sides. However, the difference between the force thresholds is not very large.

To be able to mathematically describe the models, some assumptions needed to be made. One of them is the assumption of using an offset in Stevens’ law (see Eq. 2, the offset is the $x_{0}$ term). We assumed this offset to be equal to the position of the force detection threshold. Decreasing this offset would have decreased the biases predicted from the stiffness model, while it would not have affected the predictions from the bisection model. Decreasing this term to 0 would have led to a prediction of no biases at all for the stiffness model, so the stiffness model would still have been rejected when decreasing the offset. Another assumption is that $\beta$ is 0.8. If we had chosen a much higher $\beta$, the biases predicted from the stiffness model would have been much larger than the ones expected from the force detection thresholds from the literature. So, for $\beta$ smaller and larger than 1, we can reject the stiffness model. However, when assuming both $\beta$ is 1 and $x_{0}$ is the position of the detection threshold, no distinction between the models can be made. To assess the validity of the models under these assumptions, future research using other paradigms that would be able to discriminate between the models under these assumptions would be needed, like force fields composed of nonlinear springs.

Even though our results are in accordance with previous experiments testing the idea that participants base their estimate of the center of a force field on the position(s) where the force reaches the threshold level, they are still slightly puzzling from the perspective of the sources of information that participants have. The results clearly show that participants do not use an estimate of the stiffness, even though they could have had access to this information if they wanted to, since they were exploring the force field anyway and thus were experiencing changes in position and the accompanying changes in force. By not using the stiffness information, they made large errors, which is usually something you want to avoid. This implies that force and position are not integrated in this task, even though previous research has shown that humans are able to do this (e.g., Drewing and Ernst 2006).

One explanation of this phenomenon could be that, to be able to use stiffness information, participants would have to make certain assumptions. For instance, they would need to assume that the spring is linear in the range where the forces are imperceptible. In nature, objects hardly ever behave like a linear spring, so this might explain why this assumption is not a logical one to make. However, participants should be able to assess that the part of the force field with forces above threshold level is linear, so then the assumption of a completely linear spring would not be very unlikely.

Another explanation is that stiffness information might not be a very reliable cue, since humans show poor performance in stiffness discrimination tasks (Jones and Hunter 1990). Tan et al. (1995) even argue that humans do not use stiffness information at all. They showed that, in their task of squeezing two plates in a pinch grasp, terminal force cues and work cues were the primary source of information that participants used to estimate the stiffness.

A third explanation could be that there is a cost to acquiring stiffness information, which is larger than the cost of using the strategy described in the bisection model. Since participants never received feedback about their performance, they did not know that they were making errors. If the force field would have been symmetric, the bisection model would have predicted no errors, so in that case, it could have been a smart strategy to choose. Acquiring stiffness information could be costly, because the process is very serial, so information needs to be acquired and compared over time. When only using the position where the force reaches the threshold, the task of finding that position might be of a serial nature, but the information that needs to be stored is only one or two position(s). In several studies, it has been suggested that serial strategies are more costly than parallel ones (Dopjans et al. 2012; Loomis et al. 1991). There are also examples of situations in which haptic information is neglected when it is added to a task. For example, Heuer and Rapp (2012) describe that added haptic information is neglected in a visuo-motor rotation task. In a next study, they describe a deterioration in learning of a visuo-motor rotation when augmented haptic feedback is added, so the neglect of haptic information might be a functional strategy in this task to avoid deterioration in learning (Heuer and Rapp 2014). The same process might be happening in our experiment: Even though stiffness information is provided, participants neglect it and choose the ‘easy option’ described by the bisection model to solve the task. This suggests that the brain might be able to choose if it integrates information or not, based on the possible costs and benefits of the integration. However, irrespective of which explanation is the correct one, the bisection model seems to adequately describe the outcome of the decision process of the participants.

The knowledge acquired in this experiment also has practical applications, such as for the design of haptic guidance in tele-operation applications (Abbink et al. 2012). In this technique, force fields are often used to guide the operator toward a target, which is a position at the minimum of the force field. Experiment 1 shows that it could be beneficial to add visual feedback about hand position, even if the feedback does not provide task-relevant information. Experiment 2 suggests that it might not be smart to position the target at the minimum of the force field, since operators actually hardly overshoot the target that far that they enter the part of the force field that is above threshold level behind the target. So, in practice, force fields in haptic guidance resemble a unilateral situation, in which we found large biases. Therefore, it might be better to ensure that the force is at threshold level when operators reach the target. Obviously, follow-up experiments would be needed to test whether this suggestion indeed increases operator performance.

## Conclusion

When participants are asked to estimate the center of a force field, they show a stiffness-dependent bias toward the weaker spring in an asymmetric force field. In a unilateral force field, they show a stiffness-dependent bias toward the direction of the spring. When providing visual feedback about hand position, this decreases the variability in the responses, even if it does not provide any task-relevant information. This study provides evidence against the hypothesis that participants use stiffness information to find the center of a force field. The results are in agreement with the idea that participants base their estimation of the center of a force field on the position(s) where the force reaches the threshold level, which is described mathematically in the bisection model. This shows that force and position information are not integrated in this task.
